# Pathogenesis of Nasal Polyposis: Current Trends

**DOI:** 10.1007/s12070-022-03247-2

**Published:** 2022-12-03

**Authors:** Anastasios K. Goulioumis, Konstantinos Kourelis, Magioula Gkorpa, Vasilios Danielides

**Affiliations:** 1 Department of Otorhinolaryngology, “Karamandanion” Pediatric Hospital of Patras, Erythrou Stavrou 40, 26331 Patras, Greece; 2 Otorhinolaryngology Private Practice, Mesolongi, Greece; 3 Department of Otorhinolaryngology, University Hospital of Patras, Patras, Greece; 4 Anatomy Department, School of Medicine of the University of Patras, Patras, Greece

**Keywords:** Chronic rhinosinusitis, Nasal polyps’ pathogenesis, Th 2 immune response, Endotype, Epithelial immune barrier

## Abstract

Chronic Rhinosinusitis (CRS) is characterized by edema of the sub-epithelial layers, but, only specific types of CRS are developing polyps. Nasal polyposis may develop under different pathogenetic mechanisms rendering the typical macroscopic classification of CRS, with or without nasal polyps, rather deficient. Currently, we approach nasal polyposis, in terms of diagnosis and treatment, according to its endotype, which means that we focus on the specific cells and cytokines that are participating in its pathogenesis. It appears that the molecular procedures that contribute to polyp formation, initiating with a Th-2 response of the adaptive immune system, are local phenomena occurring in the sub-epithelial layers of the mucosa. Several hypotheses are trying to approach the etiology that drives the immune response towards Th-2 type. Extrinsic factors, like fungi, Staphylococcus superantigens, biofilms, and altered microbiome can contribute to a modified and intense local reaction of the immune system. Some hypotheses based on intrinsic factors like the elimination of Treg lymphocytes, low local vitamin-D levels, high levels of leukotrienes, epithelial to mesenchymal transition (EMT) induced by hypoxia, and altered levels of NO, add pieces to the puzzle of the pathogenesis of nasal polyposis. Currently, the most complete theory is that of epithelial immune barrier dysfunction. Intrinsic and extrinsic conditions can damage the epithelial barrier rendering sub-epithelial layers more vulnerable to invasion by pathogens that trigger a Th-2 response of the adaptive immune system. Th2 cytokines, subsequently, induce the accumulation of eosinophils and IgE together with the remodeling of the stroma in the sub-epithelial layers leading, eventually, to the formation of nasal polyps.

## Introduction

The nasal cavity and sinuses’ surface are covered mainly by pseudostratified cylindrical ciliated epithelium known as respiratory. Lamina propria, the part of the mucosa deeper to the basic membrane of epithelium, is a thin layer of loose areolar connective tissue. In the course of chronic rhinitis, lamina propria becomes congested with effector cells of the immune system and cytokines, while the cell stroma undergoes tissue remodeling. The result of these alterations in the mucosa is the formation of nasal polyps covered by what macroscopically appears as “normal” respiratory epithelium.

Chronic rhinosinusitis (CRS) is a very common disease affecting almost 10% of the global population, but only 2,7% of the population is suffering from nasal polyposis [1]. Chronic rhinitis has complicated pathogenesis, not yet fully elucidated. Among others, CRS has been related to anatomic narrowing of the osteomeatal complexes and allergy. The formation of polyps, though, is a different story. On one hand, it was thought that in narrow anatomical areas in the nasal cavity, where opposing mucosal surfaces are very close, the development of polyps is more possible [2]. However, polyps still relapse even after extended surgical approaches in the lateral nasal cavity wall. On the other hand, polyposis is more possible to happen to non-atopic patients in comparison to atopic ones [1].

Every type of chronic rhinitis is characterized by some grade of mucosal edema. The fact, though, is that, typically, there is no specific amount of edema that defines a polyp. Nevertheless, only part of the cases of CRS, is concluding to such an amount of mucosal edema in order, macroscopically, for this to be characterized as a polyp. The question is what happens differently in some cases of chronic rhinitis leading them to polyposis. To make things even more complicated, not all nasal polyposis’ cases are developing under the same pathogenetic mechanism. Therefore, classifying CRS, simply according to its clinical phenotype, as Chronic Rhinitis with Nasal Polyps (CRSwNP) and Chronic Rhinitis without Nasal Polyps (CRSsNP), is rather deficient.

As basic research results are shedding light on the pathogenetic mechanisms of chronic rhinitis, characterizing nasal polyposis by its endotype becomes a new trend. So, by differentiating a CRSwNP by its endotype, meaning the cells and cytokines that are participating in its pathogenesis, our potential armamentarium can be enriched with more targeted and efficient molecular therapies, like monoclonal antibodies against cytokines [3].

### Adaptive Immune System and Formation of Polyps

The adaptive immune system is the cornerstone of nasal polyps’ pathogenesis [4, 5]. The function of the specialized adaptive immune system, which leaves immunologic memory, begins with the phagocytosis of pathogens by dendritic cells. Such pathogens can invade the sub-epithelial layers of the mucosa or can be engulfed by protrusions of the dendritic cells outside of the epithelial layer. Subsequently, dendritic cells, functioning as antigen-presenting cells, present peptides of the pathogen on their MHC II transmenbranic complexes. Recognition of the peptides by the TCR receptors of T-naïve lymphocytes activates them.

Depending on the type of the pathogen, on the additional molecules that form complexes between naïve T-lymphocytes and dendritic cells, and on the epithelial cell-deriving cytokines that can influence the lymphocytes, different transcription factors are being activated in the nucleus of the T-naïve lymphocytes leading them to potentially four different states. They may become either Th1, Th17, Treg, or Th2.

When T-naïve Lymphocytes become Th1-that secrete INF-γ-or when they become Th17-that secrete IL-17-then neutrophils are attracted in the lamina propria. Additionally, when the transcription factor FOXP3 is activated, T-naïve Lymphocytes become Treg. The role of these cells is to suppress the inflammation by secreting TGF-β. Finally, when T-naïve lymphocytes become Th2, they produce IL-4, IL-5, and IL-13. IL-4 functions as a chemotactic factor for eosinophils through upregulation of VCAM-1 in the endothelium of vascular cells [5, 6]. IL-5, on the other hand, participates in the activation of eosinophils and evasion of their apoptosis [5]. Finally, IL-13 induces B-cells that are located in follicle-like structures in lamina propria, to produce IgE locally in the mucosa [7]. The role of IgE in the polyps is to, chronically, activate Mast cells [8].

Moreover, cytokines such as IL-33, IL-25, TSLP (Thymic Stromal Lymphoprotein), eotaxin-3, and RANTES, deriving from epithelial cells after being triggered by pathogens, activate Innate Lymphoid Cells (ILC). These cells can enhance the Th2 immune response [9].

### Type of Immune Response and Polyp Formation

In the context of chronic rhinitis, when polyps are formed, among the four different types of T-helper cells, we usually detect a Th2 immune response characterized by the presence of eosinophils in the sub-epithelial layer of the mucosa. On the other hand, chronic rhinitis that is not developing polyps, but rather fibrosis of the lamina propria, is characterized by a Th1 immune response with the accumulation of neutrophils in the tissue [10]. However, there are cases of chronic rhinitis, that typically are forming polyps, like Cystic Fibrosis and CRSwNP in the Asian populations that are not characterized, typically, by a Th2 but, rather, by mixed Th1 and Th2 immune response with the accumulation of neutrophils [11]. For Asian populations, the different molecular pathophysiology is attributed both to environmental and genetic reasons. This is reflected in the observation that, the more Asian populations change their environmental factors and appear as Staphylococcus carriers, the more they appear with CRSwNP under Th-2 immune response. However, second-generation Asian patients with CRS, leaving in western countries; still have a greater chance of not forming polyps, because genetic factors skew the immune response in their mucosa towards Th-1 cytokines [12].

### Th2 Immune Response and Polyp Formation

When a Th2 immune response occurs, in the context of chronic rhinitis, in lamina propria, then several molecular reactions are taking place, including accumulation of immune cells, antibodies, and molecules that induce remodeling of the tissue stroma. These modifications are finally contributing to the development of sub-epithelial edema and eventually to the formation of nasal polyps.

A key event during a Th2 immune response is that lamina propria becomes congested with activated eosinophils [5]. These cells produce enzymes like Peroxidase and Kationic Protein which activate, subsequently, mast cells and fibroblasts. These enzymes also have a defensive-mechanism role, affecting, by oxidative stress, pathogens, but epithelial cells as well. The elevated amount of eosinophils locally in the tissue is corresponding to the high levels of eosinophils in the serum. However, it is shown that only the high amounts of eosinophils in the tissue have a prognostic significance for recurrence of the polyps. More specifically, the detection of more than 55 eosinophils per HPF in the tissue is related to recurrence [13]. Interestingly, activated Eosinophils in the serum may secondarily affect the mucosa of the lower airways. This mechanism is, possibly, explaining how nasal polyposis induces exacerbation of non-atopic asthma [14].

Additionally, during a Th-2 immune response, chemotaxis of macrophages, Basophils, Mast cells, and fibroblasts in lamina propria is taking place [15]. T-killer cells, which express CD8 + surface glycoprotein and create apoptotic FAS-ligand signaling to cells that do not express MHC-I, have been found in elevated amounts in polyps. Their role, though, still remains unspecified [16].

In the nasal polyps, a characteristic elevation of IgE is detected [7]. Interestingly, contrary to what is happening in allergic hyper-sensitivity type-I reaction, in polyps local production of IgE does not correspond to elevated amounts of IgE in the serum. Therefore, allergic tests, like Prick tests are typically negative in the case of idiopathic nasal polyposis [17]. It is noteworthy that, high level of IgE in the tissue is not proven to be valuable as a prognostic factor for polyposis recurrence [18]. Finally, IgG and IgA autoantibodies have been detected in the polyps. These are anti-nuclear antibodies, similar to those found in lupus erythematosus, which are acting against epitopes on the epithelial cells [19].

Tissue stroma remodeling in the lamina propria occurs, mainly, by the formation of a net created by Fibrin, produced by fibroblasts, and FXIII-A factor, produced by macrophages. This net can be, potentially, dissolved by t-Pa that is produced by epithelial cells. However, enhanced IL-13 production in a Th-2 response inhibits t-Pa [20]. Epithelial cell-derived IL-25 can transform fibroblasts to myofibroblasts which express α-sma and vimentin [21]. These cells secrete metalloproteinases, like MMP-9 and MMP-2, which can also contribute to stromal remodeling [22]. Interestingly, stroma remodeling is responding to medications that have an MMP inhibitor effect like doxycycline [15]. Another metalloproteinase, MMP-13 is responsible for the replacement of normal collagen type IV by collagen type II and V in the basic membrane. This makes the basic membrane thicker [15]. On the other hand, though, polyps’ stroma is characterized by scarcity of collagen due to low levels of the pleiotropic factor, TGF-β. Low TGF-β level is attributed to the paucity of Treg lymphocytes in the mucosa in the context of a Th2 immune response [10].

A mature polyp is characterized by a distribution of immune cells all over its mass, contrary to newly formed polyp, where immune cells accumulate only in the upper levels close to the epithelial cells [15]. A mature polyp is, additionally, characterized by pseudocysts formation under the action of MMP-9 [22]. Higher permeability of vessels owning to histamine, PDGF, and C3a factor of the complement is related to the high amounts of albumin in the tissue stroma [23, 24]. There are references of high amounts of hyaluronic acid of high molecular weight probably due to polymorphisms in hyaluronic acid synthase or polymorphisms in hyaluronidases [25]. Both albumin and abnormal hyaluronic acid, creating an osmotic environment, contribute to the development of edema in the lamina propria and eventually to polyp formation.

### What is Inducing Th2 Immune Response in the Lamina Propria

As was previously discussed, the types of Chronic Rhinitis in which a Th2 immune response is taking place are concluding to the formation of polyps. The question, though, is which are the conditions that induce a Th2 response. There are extrinsic and intrinsic hypotheses. The extrinsic hypotheses are related to pathogens, allergens, and toxic compounds that can skew the immune response towards a Th2-type. The intrinsic hypotheses are based on an exaggerated reaction of the immune system against the aforementioned pathogens or an autonomic reaction of the immune system due to polymorphisms of specific genes.

### Fungus Hypothesis

This hypothesis is based on the fact that fungal species are detected in almost all cases of nasal polyposis. There are references that eosinophils from patients with polyposis are reacting against a fungal species called Alternaria, but such a reaction is not detected with eosinophils of controls [26]. Additionally, allergic fungal rhinitis, a chronic condition accompanied, in almost 100% of the cases, by polyps, is a well-described allergic hypersensitivity type-I reaction towards fungus [27]. This type of Chronic Rhinitis, which does not usually co-exist with asthma, is characterized by eosinophilic mucin similar to nasal idiopathic polyposis, but with the additional detection of fungal species in the mucin. Allergic Polyposis attributed to fungus is reaching the percentage of 14% among different types of CRSwNP.

Yet, against this hypothesis is the fact that fungal species can be evenly detected in the vast majority of CRS that is not characterized by polyps. Additionally, neutrophils and not eosinophils are the specific immune cells against fungus. Moreover, idiopathic CRSwNP is not an atopic, type-I, allergic hypersensitivity reaction, since nor high IgE is detected in the peripheral plasma nor allergic tests are typically positive. Interestingly, it is more typical to find nasal polyps in a non-atopic patient in comparison to an atopic one. Only 5% of atopic patients appear with polyps and only 13% of patients with CRSwNP have positive RAST test. Finally, anti-fungal therapy is not proven significantly effective against nasal polyposis [28].

So, the fungal hypothesis has been abandoned as the main theory for nasal polyposis’ formation. However, fungus can trigger epithelial cells to produce IL-33 which activate Innate Lymphoid Cells (ILC) to produce Th2 cytokines, contributing in such a way to the polyposis’ pathogenesis [29].

### The Hypothesis of Biofilms and Superantigens

Pathogens like staphylococcus can be found in Biofilms covering the mucosa. The biofilm consists of an extracellular matrix that is composed of polymeric substances, such as polysaccharides. Microbes that are found in Biofilms are protected from immune system insult and antibiotics. Some microbes that can return in their free planktonic form are capable of producing exotoxins that function as superantigens. These kinds of antigens activate a great amount of T-naïve lymphocytes with a non-canonical TCR—antigen—MHC II interaction. Superantigens are rather bridging the MHC-II molecules of the antigen-presenting cells with the TRC of naïve T-Lymphocytes. These conditions lead to massive production of Th1 and Th2 cytokines and subsequently to the formation of polyps [30]. Interestingly, in the polyps where there is high production of IL-5, the pathognomonic Interleukin of a Th2 immune response, we also detect local IgE against staphylococcus [31]. Special IgE against staphylococcus can activate the factors of the complement in the mucosa [23]. Moreover, staphylococcus induces secretion of IL-6 by epithelial cells and activates factor HIF-1α, which may induce hypoxia and thus promote polyps’ formation [32]. An additional indication for the role of staphylococcus in the pathogenesis of polyposis is derived from statistics. In the general population, 20% of the population are carriers of staphylococcus in their nasal cavities. In the special population of people with nasal polyps, staphylococcus can be detected in 60% of the cases, while when polyposis is combined with asthma then 87% of the patients have staphylococcus in their nose [33].

Nonetheless, biofilm without the presence of superantigens does not make any difference to the preponderance of chronic rhinitis to form polyps [34]. Superantigens finally, can be found in cases of CRS without Nasal polyps, indicating that even though they contribute to polyposis’ pathogenesis, they cannot be considered as the main pathogenetic mechanism of this phenomenon.

### The Hypothesis of Microbiome

The nasal cavity is an anatomic area that hosts normal flora consisting of various microbes. The particular microbiome, each individual develops, relies on genetic together with environmental factors, like the type of microbes to which a person was exposed in the early stages of life [35]. Patients suffering from Chronic Rhinosinusitis have a different microbiome when comparing it to controls. However, with the exception of Cystic Fibrosis, there is no single type of microbiome corresponding to every specific nasal polyposis subtype [36]. Medications that we use in CRS do influence nasal flora in the long term. Therefore, the microbiome hypothesis applies, mostly, in the case of Cystic Fibrosis where we can detect a poorer variety of microbes, nevertheless characterized by high bacterial load [36]. It is noteworthy, though, that sort-term antibiotic or steroid usage imposes only slight alteration to nasal microbiome [37]. Finally, unsuccessful attempts to treat CRSwNP with probiotics reflect the indirect role of the microbiome in nasal polyposis pathogenesis. This comes in line with observations in autoimmune Crohn’s disease. This condition appears with similarities, in terms of pathophysiology, with CRSwNP. It shows, also, little response to therapies with probiotics [38, 39].

### The Hypothesis of Vitamin D

Vitamin D deficiency is lately related to CRSwNP pathogenesis through its influence on the immune system. Interestingly, it has been suggested that vitamin D deficiency is more common in the cases of CRS with polyps compared to CRSsNP [40]. Vitamin D enhances the antibacterial action of macrophages. In the case of vitamin D deficiency, epithelial cells produce RANTES and IL-6 which trigger the Internal Lymphoid Cells (ILC) to produce Th-2 cytokines. Additionally, vitamin D deficiency is related to fibroblasts’ accumulation in the lamina propria and enhanced activation of Th-2 cells by the antigen-presenting dendritic cells [41]. There is a vivid interest in the literature for the role of vitamin-D deficiency in polyposis. This is even further supported by the observation that in the case of asthma, vitamin D can make steroid therapy more effective [42]. Interestingly, vitamin D is also produced locally in the tissues. An intrinsic hypothesis for the pathogenesis of polyposis has emerged with the detection of polymorphisms in the genes that encode 1-α-hydroxylase. This can lead to a local deficiency of vitamin D and, subsequently, to the formation of nasal polyps [43].

### The Hypothesis of Treg Lymphocytes

According to another intrinsic hypothesis, polymorphism of the transcription factor, FOXP3 that transforms the T-naïve lymphocytes to Treg is leading to less Treg activity in the mucosa [44]. Normally, Tregs are inflammation suppressor cells that can inactivate T-killer lymphoid cells by secreting IL-10. Moreover, the low presence of Treg cells is leading both to enhanced Th2 immune response and to lower levels of TGF-β which, eventually, leads to polyp formation. Finally, it is shown that T-naive lymphocytes are skewed to transformation to Th-17 instead of Treg under the influence of IL-6 deriving from the epithelial cells in the context of staphylococcal presence on the mucosa [45].

### The Hypothesis of Nitric Oxide

This is an intrinsic hypothesis related to Nitric Oxide Synthases (NOS) polymorphism [46]. The NOS is mostly found on the cilia of the epithelial cells. Low levels of NO in the mucosa are implicated with reduced activity of the cells of the innate immune system against pathogens, especially Pseudomonas. Additionally, a low level of NO is related to the reduced biting rate of epithelial cells’ cilia. These can alter the nasal microbiome skewing adaptive immune response to Th-2 cytokines and eventually to polyps’ formation [47].

### The Hypothesis of Eicosanoids

Polymorphisms of the enzymes that metabolize arachidonic acid in Mast cells, like cyclooxygenase and lipoxygenase, can induce enhanced production of leukotrienes that activate eosinophils [48]. Additionally, leukotrienes can trigger epithelial cells to secrete IL-6 that leads an immune response of Th-2 type [49]. According to an extrinsic hypothesis staphylococcal superantigens can enhance leukotriene expression under the presence of high IL-4 in the tissue [50].

It is questionable, though, whether altered metabolism of Eicosanoids is a pathogenetic mechanism that applies to every subtype of CRSwNP or if it characterizes only a specific type of CRS with polyps known as AERD (Aspirin Exacerbated Respiratory Disease). AERD affects 2,5% of the general population. It is noteworthy that in the sub-population of patients with nasal polyps and non-atopic asthma, 40% have sensitivity to aspirin. AERD is a polyposis that combines non-atopic rhinitis, asthma, and sensitivity to aspirin and NSAIDs. The genetic base of this type of CRSwNP, attributed for instance to HLA variability, is reflected in the fact that AERD is hereditary in 42% of the cases [51]. Detection of Eicosanoids’ metabolism problems in nasal polyposis patients enables physicians to broaden their therapeutic options with suppressors of lipoxygenase, desensitization of aspirin, and antileukotriene drugs [52].

### Hypoxia Hypothesis

Chronic rhinitis can create a hypoxic environment for the mucosa in several ways. Edema of the mucosa around the osteomeatal complex of the middle meatus forms a hypoxic state for the epithelial covering of the sinuses [53]. Basically, even the edema of the lamina propria, where the vessels of the mucosa are located, makes the blood supply to the epithelial layer less efficient. Thus, epithelial cells of an edematous mucosa are functioning, eventually, in a hypoxic state.

Decreased availability of oxygen in the cellular environment induces the expression of a type of transcription factors which are known as Hypoxia-Inducible Factors (HIF). A member of the HIF family, HIF1-a, is implicated in the molecular phenomenon called epithelial to mesenchymal transition (EMT). More specifically, HIF 1-a increases the transactivity and expression of a nodal transcription factor for EMT, known as ZEB-1 [54].

Epithelial to mesenchymal transition (EMT) is a molecular phenomenon that explains how epithelial cells develop migratory capacity by abolishing some epithelial characteristics and acquiring mesenchymal phenotypical and molecular traits [55]. This process is adequately described in embryogenesis and has applications in the healing of epithelial tissues, inflammation, and carcinoma metastasis. In the course of EMT, epithelial cells lose their polarity, abolish their cilia and downregulate E-cadherin leading to impairment of the epithelial barrier. Additionally, in the context of this transformation, metalloproteinases’ (MMPs) expression is also enhanced. So, ΕΜΤ by harming the epithelial immune barrier, the first line of defense of mucosa against pathogens, may lead to an enhanced reaction of the immune system, concluding to polyps’ formation [56].

### The Hypothesis of Epithelial Immune Barrier

Bernstein in 1994 had expressed the idea that improper airflow in the nasal cavity injures the epithelial cells leading to exposure of sub-epithelial layers [57]. Subsequent excessive reepithelization can create a polyp [57]. The most current trend, for the pathogenesis of nasal polyposis approach, is the epithelial immune barrier hypothesis which combines the intrinsic and extrinsic theories. According to this theory, a defective epithelial barrier and a dysfunctional repair system of damaged mucosa, render the sub-epithelial layer- lamina propria- more vulnerable to invasion by pathogens. These, subsequently, induce a local Th-2 immune response which is followed by the molecular cascade of events that lead to a polyp formation [58]. Interestingly, local steroids, together with their inflammation-suppressive role, contribute to the repair of the epithelial layer [59]. A normal epithelial barrier consists of the normal presence and function of mucus, epithelial cilia, adhesion molecules, and antimicrobial substances.

Mucus has a role as a first-line defense mechanism by trapping pathogens. For the proper clearance of mucus, both its appropriate viscosity and the normal function of the epithelial cells’ cilia are necessary. Cystic Fibrosis is a genetic disease that is characterized by mutations of the gene that encodes a chloride channel that is responsible for the homeostasis of mucus viscosity. In 70% of the cases, there is a specific mutation, ΔF 508, of the CFTR gene. In the rest 30% of the cases, though, there can be various other mutations of this specific gene for which a patient with idiopathic nasal polyposis can be heterozygous [60].

Additionally, some conditions lead to dysmotility of epithelial cilia. These can be genetic, like mutations of the gene that encodes dynein in Kartagener’s syndrome. Another example is the polymorphisms of bitter taste receptors located on the cilia, which interfere with ciliary motion [61, 62]. Moreover, there are secondary environmental factors, which influence ciliary function, like those associated with low levels of oxygen or NO [63]. So, thick mucus or improper function of cilia render clearance of the mucus inefficient and eventually alter the microbiome and its effects on the adaptive immune system. Overall, 68% of patients with cilia dysmotility diseases are suffering from CRS with polyps [64].

Epithelial cells express on their lateral surfaces, adherence junctions that reserve adhesion of one cell to its adjacent cells. Complexes like desmosomes, zona occludens, and tight junctions are comprised of adhesion molecules such as cadherins and occludin. Cytokines that are released during a Th-2 immune response, like oncostatin-M and IL-13, can dismantle adherence junctions [65]. The creation of anti-ds DNA autoantibodies in the polyps may also contribute to a leaky immune barrier [19]. A similar effect, finally, occurs in the course of the Epithelial to Mesenchymal Transition phenomenon (EMT) where downregulation of the expression of E-cadherins constitutes a key event. With the partial damage of adhesion, the physical epithelial barrier becomes leaky, rendering the adaptive immune system, in lamina propria, more exposed to pathogens [56].

Finally, epithelial cells produce and secrete antimicrobial substances like lysozyme, lactoferrin, and S-100. Polymorphisms of the genes that encode these proteins or even epigenetic modifications, like DNA methylation of these genes, may lead to an altered nasal microbiome that becomes more reactive to the immune system [58]. Similarly, polymorphisms of the Pattern Recognition Receptors (PRRs) of macrophages can make the innate immune system, the first defense line, less effective and subsequently can skew the adaptive immune system towards a Th-2 immune response [66]. Antimicrobial substances, on the other hand, may contribute to the creation of a leaky barrier. Interestingly, high expression of IL-5 in the mucosa is forcing eosinophils to produce cystic formations that contain mitochondrial DNA and cationic proteins which function as extracellular traps. These have an antimicrobial action. At the same time, though, a side effect damaging the epithelial barrier can not be avoided [67].

## Conclusion

Research is gradually shedding light on the pathogenesis of nasals polyposis. Currently, we can characterize a polyposis’ case by its specific endotype and, thus, approach its treatment with molecular therapies targeting nodal cytokines. Monoclonal antibodies against IL-5, IL-4, and IL-13 are already in use [3]. Combined administration of monoclonal antibodies can have a better result, considering that anti-IL-5 reduces eosinophil title in the serum impressively, but not to the same degree in local tissues. This happens due to the existence of additional epithelial cell-deriving cytokines with a chemotactic role, for eosinophils, in the mucosa. Another promising therapeutic approach is the use of anti-IgE monoclonal antibodies. This prevents IgE from binding to its receptor on Mast cells but in the long term, it leads also to downregulation of the receptors [58]. Other novel therapeutic approaches are developed based on the elucidation of the pathogenetic mechanisms of polyposis. Proton-pumps inhibitors show, also, an inhibitory action against the H/K ATPase of the epithelial cells, preventing them from secreting eotaxin-3 [68]. Similarly, in the eosinophilic esophagitis, a condition with analogous pathophysiology with CRSwNP, PPIs are reducing eosinophil amounts even in the proximal part of the esophagus which is not under the direct influence of acid reflux [69]. Additionally, Angiotensin II blockers show a positive effect in nasal polyposis, possibly, through inhibition of periostin [70]. This is a cytokine that is expressed in epithelial cells under the influence of IL-13. It has a signaling role for epithelial to mesenchymal transition that leads to damage of the epithelial barrier of the mucosa. Yet another interesting therapeutic approach is with inhibitors of the macrophage migration inhibitory factor (MIF) which is a pro-inflammatory mediator able to antagonize the inhibitory effects of glucocorticoids on the expression of IL-6 [71]. Finally, transmembrane P-glycoprotein, which is expressed in the epithelial cells and act as a xenobiotic pump, has been shown to enhance Th-2 immune response and eosinophil accumulation in the mucosa. In line with these, the inhibitor of calcium pumps, verapamil, is shown to inhibit P-glycoprotein as well [72].

Summing up, the idiopathic nasal polyposis in western populations does not seem to have a typical allergic background. Molecular procedures that contribute to polyp formation, initiating with a Th-2 response of the adaptive immune system, are local phenomena occurring in the sub-epithelial layers of the mucosa. Several theories are explaining the etiology that skews the immune response towards Th-2. Extrinsic factors like pathogens, fungus, staphylococcus superantigens, biofilms, and altered microbiome can contribute to a modified and intense local reaction of the immune system. Some theories based on intrinsic factors like the elimination of Treg lymphocytes, low local vitamin–D levels, high levels of leukotrienes, hypoxia with epithelial to mesenchymal transition, and altered levels of NO, add pieces in the puzzle of the pathogenesis of the polyps. Finally, the most current theory that combines all the other pathogenetic mechanisms is that of the epithelial immune barrier dysfunction. According to this, intrinsic and extrinsic conditions can damage the epithelial barrier rendering sub-epithelial layers more vulnerable to invasion by pathogens that trigger a Th-2 type response of the adaptive immune system. Th-2 cytokines, subsequently, orchestrate all the molecular phenomena that conclude to the polyps’ formation.

## Declarations

### Human or Animal Participants

This article does not contain any studies with human participants or animals performed by any of the authors.
